# Health-related quality of life and its associated factors among infertile women compared with fertile women in public hospital Addis Ababa, Ethiopia: a comparative cross-sectional study

**DOI:** 10.1186/s12905-024-03163-3

**Published:** 2024-07-23

**Authors:** Biniam Yohannes Wotango, Bezatu Mengiste, Samrawit Solomon

**Affiliations:** 1 Clinical Governance and Quality Improvement Directorate, Gandhi Memorial Hospital, Addis Ababa, Ethiopia; 2 School of Public Health, St. Paul’s Hospital Millennium Medical College, Addis Ababa, Ethiopia

**Keywords:** Quality of Life, WHOQoL-BREF, Infertile Women, Addis Ababa, Ethiopia

## Abstract

**Background:**

Infertility can have detrimental physical, psychological, and social effects that significantly impact health-related quality of life. Although the impact of infertility on quality of life is well established, there is a lack of research comparing the quality of life between fertile and infertile women in Ethiopia.

**Methods:**

A hospital-based comparative cross-sectional study was conducted among 287 infertile and 301 fertile women. Participants were selected using systematic random sampling. A structured, validated tool was used to collect data. An independent sample t-test was conducted to determine if there was a difference in the study participants' quality of life domains and the mean total quality of life score. Multiple linear regressions were used to correlate quality of life scores with significant predictor factors for the infertile group.

**Results:**

Infertile women had a mean total Herbal of 66.54 ± 10.18, and fertile women (72.68 ± 7.57) were found to be statistically different between the groups. All domains except the physical domain were significantly different between the groups. Duration of marriage (β = -0.529), number of previous sexual partners (β = -0.410), total number of working hours per day (β = -0.345), types of infertility (β = -0.34), and history of the sexually transmitted disease (β = -0.277), in decreasing order of effect, were found to be associated with the quality of life of infertile women (R^2^ = 0.725).

**Conclusions:**

The study found that infertile women had a lower mean HRQoL score compared to fertile women, with all domains except for the physical domain being significantly different between the two groups. This suggests that infertility can have a significant impact on various aspects of a woman's life, including her emotional well-being, social functioning, and psychological health. The factors associated with the quality of life of infertile women were the duration of marriage, the number of previous sexual partners, the total number of working hours per day, the types of infertility, and the history of sexually transmitted diseases, with duration of marriage having the strongest association. These findings highlight the need for healthcare providers to address the psychological and social aspects of infertility.

**Supplementary Information:**

The online version contains supplementary material available at 10.1186/s12905-024-03163-3.

## Background

The World Health Organization (WHO) defines quality of life (QoL) as an individual’s perception of their position in life in the context of the culture and value systems in which they live and with their goals, expectations, standards, and concerns [1]. It is a broad-ranging concept affected in a complex way by the person's physical health, psychological state, level of independence, social relationships, personal beliefs, and their relationship to salient features of their environment [2]. The quality of life-related to health (QoL) is now considered a key tool for measuring infertility in infertile women [3]. Due to the various adverse physical, psychological, and social effects of infertility, the evaluation of QoL components may lead to the identification of various aspects of lifestyle and help them plan a better treatment [4].

Globally, 10–15% of couples face infertility, affecting millions seeking to conceive. Beyond physical challenges, it induces mental health disorders, strained relationships, and reduced self-esteem [5]. Studies consistently reveal lower quality of life in the infertile, impacting emotional well-being, social functioning, and overall life satisfaction compared to their fertile counterparts [6].

According to a WHO report in 2021, globally, infertility affects 10%–15% of couples of reproductive age, of which 30% are living in sub-Saharan Africa and South Asia; this makes it one of the most common diseases for people between the ages of 20 and 45 [7]. The negative impact of infertility on quality of life (QoL) has been well documented in some countries. For example, in Middle Eastern and African countries, infertility is a major concern for couples and can cause extreme psychological consequences, especially for women [8, 9].

In recent years, infertility has been rising in many countries around the world, including Ethiopia [10]. In Ethiopia, the prevalence of primary infertility ranges from 1.4 to 9.0%, whereas the prevalence of secondary infertility ranges from 2.5 to 15.1% [11, 12]. In Addis Ababa, this number of affected couples by infertility reaches up to 27.8%, and it has been reported to negatively affect the quality of life (QoL) of women [13].

Several studies have consistently shown that infertility is associated with decreased scores (poor) in QoL domains affecting mental health, emotional behavior, psychological, physical, environmental, vitality, and social domains [14]. There are numerous studies conducted in developed countries assessing the QoL of infertile women using different internationally validated tools, but since most of the studies on the QoL of fertile and infertile women were conducted in developed countries, much is not known about the topic in developing countries, so there is a knowledge gap in the QoL of infertile women and its difference from the QoL of fertile women, as well as associated factors [15]. While infertility treatments are successful in a considerable proportion of cases, they often hurt the patient's quality of life. Hormone treatments may have various psychological side effects and can be time-consuming and stressful, further contributing to the overall burden of infertility [16].

Therefore, this study aims to fill the gap in the literature by exploring the QoL of infertile women on follow-up at infertility clinics and fertile women attending outpatient departments of public hospitals for family planning services in Addis Ababa, Ethiopia. It will examine the factors that contribute to the QoL of infertile women, including socioeconomic status, duration of infertility, marital satisfaction, and reproductive history. The scope of this study is limited to women attending public hospitals in Addis Ababa, Ethiopia, and it will provide a basis for further research in this area.

## Methods

### Study setting, study design, and period

A facility-based comparative cross-sectional study was conducted from May 15, 2022, to August 15, 2022, in selected public hospitals in three of the three public infertility clinics located in Addis Ababa, Ethiopia, a developing country located in East Africa. All three hospitals were chosen purposefully for this study, namely Gandhi Memorial Hospital Saint Paul Hospital Millennium Medical College, and Tikur Anbessa Specialized Hospital.

### Participants

#### Source population

All clients with infertility problems visiting the public hospitals’ outpatient infertility clinic and all clients without infertility problems visiting the family planning clinic in the same hospitals were the source population.

#### Study population

Selected women diagnosed with infertility who came to the outpatient infertility clinic and selected clients without infertility problems visiting the family planning clinic in the same hospitals during the study period.

#### Study unit

Individuals with infertility who presented to the infertility clinic and individuals without infertility problems presented to the family planning clinic for family planning service.

### Eligibility criteria

#### Inclusion criteria

- Married Women in the age range of 15–49 years diagnosed to have primary or secondary infertility on follow-up in infertility clinics in public hospitals during study sampling
- Married women, or separated but in union in the age range of 15–49 coming to the outpatient department for family planning service and having at least one child (comparison group).

#### Exclusion criteria

- Women having known mental illnesses (diseases under supervision or treatment by a psychiatrist or psychologist) that are not related to infertility.
- Women have suffered from the experience of disastrous or adventurous events in the last three months.
- Women having known physical problems (spinal cord injury, amputation, paralysis, and deformity of the limb), medical diseases (cardiovascular diseases, lung disease, hyperthyroidism, hypothyroidism, epilepsy, and diabetes), gynecologic other system cancer, or other unmentioned chronic illness.

### Sample size determination and sampling technique

The sample size was estimated using the sample size calculation formula for comparison between two means for equal sample sizes by using the significant mean difference for the two groups and taking the one with the highest number. Therefore, taking the highest final sample size, the final calculated sample size became 690 for both groups (345 for infertile women and 345 for the comparison group). The sample size was taken from all three hospitals and was allocated proportionally to each hospital. Women with infertility and women without fertility problems were selected from TASH, GMH, and SPHMMC using a systematic random sampling method. The sampling interval (K) was approximately 7 for all three hospitals. Using the first random number, the 4th patient was chosen at TASH (resulting in every 11th patient being included), the 2nd patient was selected at GMH (equating to every 9th patient being included), and the 5th patient was chosen at SPHMMC (resulting in every 12th patient being included).

### Study procedure

Every clinic session started with an informative speech from the researcher detailing the goals of the study, the inclusion and exclusion criteria, and potential advantages. Next, a systematic random sampling process was used. After obtaining a list of all the eligible women who visited the clinic every day, research assistants approached each one of them about taking part in the study. Women who agreed to participate were provided with appropriate privacy and confidentiality. The researcher and research assistants administered a structured questionnaire to each participant individually, away from the hearing of others. Before distributing the questionnaire, informed consent was acquired from each participant in a language they could understand. The participants were required to respond to each question they were asked about individually.

### Operational and term definitions

#### Term definitions

- **Quality of life (QoL):** is defined as 'individuals' perception of their position in life in the context of the culture and value systems in which they live and with their goals, expectations, standards, and concerns. Quality of life in the context of my study is health-related quality of life (HRQoL) and is measured in four domains or subscales. If there is at least a significant difference between one of the domains or subscales, then we say there is a difference in quality of life between the infertile and fertile groups in my study [17].
- **Infertility:** Infertility is” the inability to conceive after 12 months of unprotected regular sexual intercourse." And includes primary infertility and secondary infertility [18].
- **Primary infertility:** Primary infertility is a condition in which a couple has been married for at least one year and hasn't achieved conception despite having regular, unprotected sexual intercourse [18].
- **Secondary infertility:** a condition in which a couple who had at least one previous conception, irrespective of the outcome, was trying to conceive for the last 12 months or more regular sexual intercourse [13]**.**
- **Dysmenorrhea:** Pain associated with menstruation [19].

### Operational definitions

- **Health-related quality of life (HRQoL):** an individual’s or group’s perceived physical health, social relationships, psychological health, and environmental health of an individual over time [20].
- **Total mean HRQoL:** the mean of the four WHOQoL BREF domains in my study [21].
- **Comparison group or fertile women:** married women aged between 15 and 49 years who come to the outpatient department for family planning service and have at least one child [22].

### Tools and measurement

The study was conducted using the abbreviated World Health Organization Quality of Life questionnaire and an author-constructed questionnaire after reviewing relevant literature. The author-constructed questionnaire contains items concerning socio-demographic data, gynecological history, obstetrics history, and sexual history. The primary version of the questionnaire was pretested in 5% of the total sample size (35) in a private infertility center called Alhikima Center, which is located in Bole. Using the information from the pretesting study, the respondents' comments and concerns were modified. The WHOQOL-BREF questionnaire contains 26 items that measure the following domains: physical health (7 items), psychological health (6 items), social relationships (3 items), and the environment (8 items). This questionnaire also includes two other items to evaluate health status and quality of life generally. Each question is provided according to the 5-point scale, from 1 to 5. The scoring of the questions has a positive direction, i.e., the higher the number of scores, the higher the quality of life, except for questions 3, 4, and 26, the higher values of which evidence a lower quality of life. Therefore, the scores of these items were then reversed into positive scores. The scoring for domains was established by calculating the raw score for questions belonging to a given domain. Each item of the WHOQOL-BREF is scored from 1 to 5 on a response scale, which is stipulated as a five-point ordinal scale. The scores were then transformed linearly to a 0–100 scale, and the result for each domain was transformed to 100 [17]. Higher transformed scores on each of the domains indicate a higher quality of life in that particular area (i.e., someone who scores 75 on the social relationships domain has a higher perceived quality of life with social relationships than someone who scores 25) [23].

### WHOQOL-BREF measurement validity and reliability

The validity and reliability of the Amharic version of the abbreviated World Health Organization Quality of Life instrument (WHOQOL-BREF) were assessed in patients with diagnosed type 2 diabetes at Felege Hiwot Referral Hospital in Bahir Dar in 2019. Cronbach's alpha coefficients for physical, psychological, social, and environmental domains were 0.84, 0.74, 0.58, and 0.71, respectively. The overall finding of the analysis implies that the Amharic version of the abbreviated World Health Organization Quality of Life instrument has internal consistency and validity to investigate the quality of life and can be used for studies that are going to be conducted in Ethiopia [24]. The data was collected through face-to-face interviews and self-administered from the study participants by 1 MSC nurse, 2 midwives who hold BSc degrees, and 2 general practitioners, and was supervised by the principal investigator. In addition to this, a close follow-up by the principal investigator was done.

### Data quality assurance

The quality of the data was controlled starting from the time of questionnaire preparation. The tool has two parts: WHOQOL-BREF and the author's questionnaire. The author's questionnaire was developed by reviewing relevant literature on the subject to ensure reliability. First, the questionnaire, which was prepared in English, is translated into Amharic. To ensure the consistency of the tool, it was translated back into English. The training was conducted for data collectors and supervisors on the purpose of the study and the procedures of data collection one day before the study. They were also aware that each day there would be supervision for data collectors. After completing the training, trainees conducted a pre-test using 5% of the total sample size (35) at a private infertility center called Alhikima. Using the information from the pretesting study, the respondents' comments and concerns were modified to make the tool easier and avoid variation in understanding. A close follow-up by the principal investigator was done. During data collection, the supervisor continually received questionnaires from data collectors and reviewed them for completeness, accuracy, and consistency daily. Incomplete, inconsistent, and invalid data were refined properly to get the maximum quality of data before, during, and after data entry.

### Data processing and analysis

The collected data were first checked for missing values and outliers. Missing values were replaced using an imputation method (mean, median, or mode), and outliers were identified using a box plot that was either removed or adjusted. The collected data were cleaned and entered directly during data collection. Data were entered into Epi Info Version 7.2 and SPSS by three generals and then exported to Statistical Package for Social Studies (SPSS) Version 25. Descriptive data analysis was performed using means, medians, interquartile range, and continuous variables expressed in mean and standard deviation, and the mean score of quality of life was compared using independent group t-tests and one-way ANOVAs. A linear regression model was used to examine the relationship between the dependent variable and one or more independent variables. The dependent variable was continuous, and the independent variables were either continuous or categorical. The model was tested for assumptions, including linearity, normality, homoscedasticity, and independence of residuals. A suitable regression model was selected based on the research question and the statistical significance of the independent variables. Regression models, including simple linear regression and multiple linear regressions, were considered. The coefficients of the regression equation were estimated using the least-squares method. The regression equation was then used to predict the value of the dependent variable for different values of the independent variable. The regression model was validated by checking the assumptions of linearity, normality, homoscedasticity, and independence of residuals. R-squared, adjusted R-squared, F-statistic, and p-value statistical tests were performed to check the model's goodness of fit. In addition to statistical significance, effect size statistics were also calculated to determine the practical significance of the independent variables in explaining the variance of the dependent variable. The effect size statistics included the beta coefficient, which represents the change in the dependent variable for a one-unit change in the independent variable, and the R-squared, which represents the proportion of variance in the dependent variable explained by the independent variable (s). The coefficients of the regression equation were interpreted to determine the direction and magnitude of the effect of the independent variable on the dependent variable. The standardized beta coefficient, which represents the change in the dependent variable for a one-standard-deviation change in the independent variable, was used to compare the effect of different independent variables on the dependent variable. Alternatively, unstandardized beta coefficients could be used to interpret the effect of the independent variable on the dependent variable on the original scale. A confidence limit of 95% and a *p*-value ≤ 0.05 were used as a cut-off point to determine the presence of statistical significance. Microsoft Excel 2019 was used to make graphs and charts. Tables, figures, charts, and texts are used for data presentation.

## Result

### Socio-demographic characteristics of the study participants

Socio-demographic characteristics of the study participants. A total of 588 participants (287 infertile and 301 fertile women) were included in the study, giving an 86.2% response rate. Fifty-eight infertile and forty-four fertile clients were not included due to incomplete responses, missing important data from the charts, and unwillingness to be included in the study. To check for the adequacy of the sample size, post hoc power analysis was done to determine the power of the study using G Power version 3.1.9.4 for both the descriptive and the regression parts. The mean age of infertile women was 30.98 years with ± 3.58 standard deviations (SD), and that of fertile women was 30.43 years with ± 3.74 standard deviations (SD). The socio-demographic characteristics of the study participants are summarized in Table 1.

**Table 1:** Socio-demographic profile between infertile women and fertile women in public hospitals, Addis Ababa, Ethiopia

Variable	Response	Infertile women (*N* = 287) Frequency(percentage)	Fertile women (*N* = 301) Frequency(percentage)
Age of respondent (in years)	Mean ± SD	30.98 ± 3.58	30.43 ± 3.74
	< 35	243 (84.7)	270 (89.7)
	≥ 35	44 (15.3)	31 (10.3)
Age at marriage (in years)	Mean ± SD	25.66 ± 2.86	24.54 ± 2.80
	< 25	136 (47.4)	191 (63.5)
	≥ 25	151 (52.6)	110 (36.5)
Duration of marriage (in years)	Mean ± SD	5.18 ± 2.29	6.13 ± 3.33
	< 5	198 (69)	144 (47.8)
	≥ 5	89 (31)	157 (52.2)
Total household income (in birr)	Mean ± SD,	5021.3 ± 1676	6888.9 ± 2127.8
	IQR	(2500–9100)	(1500–15000)
	Low (< 2500)	11 (3.8)	3 (1)
	Middle(2500–7500)	233 (81.2)	168 (55.8)
	High(≥ 7500)	43 (15)	130 (43.2)
working hours per day	Mean ± SD	7.84 ± 1.62	8.53 ± 1.93
	< 8	240 (83.6)	189 (62.8)
	≥ 8	47 (16.4)	112 (37.2)
Place of residence	Urban	276 (96.2)	278 (92.4)
	Rural	11 (3.8)	23 (7.6)
Marital status	Married	287 (100)	252 (83.7)
	Separated	0 (0)	49 (16.3)
Educational status of women	Read and write	0 (0)	37 (12.3)
	Primary education	56 (19.5)	84 (27.9)
	Secondary education	105 (36.6)	97 (32.2)
	College/University	126 (43.9)	83 (27.6)
Educational status of husband	Read and write	0 (0)	11 (3.7)
	Primary education	11(3.8)	25 (8.3)
	Secondary education	68 (23.7)	131 (43.5)
	College/University	208 (48.8)	134 (44.5)
Occupation of the women	Private employee	39 (13.6)	67 (22.3)
	Housewife	104 (36)	141 (46.8)
	Governmental employee	142 (49.5)	90 (29.9)
	NGO	2 (0.7)	3 (1)
Occupation of the husband	Private employee	24 (8.4)	102 (33.9)
	Daily labourer	40 (13.9)	27 (9)
	Governmental employee	221 (77)	166 (55.1)
	NGO	2 ( 0.7)	6 (2)

### Past obstetrics and sexual characteristics of the study participants

About 301(100%) of the fertile group and 56 (19.5%) of the infertile groups had a previous history of pregnancy with a mean number of 1.0 for infertile women and 2.68 ± 1.2 for fertile women. Regarding the previous number of sexual partners, the infertile group had a 2.51 mean number of sexual partners with ± 0.94 standard deviations (SD), and the fertile group had a 2.32 mean number of sexual partners with ± 0.80 standard deviations (SD). About 122 (42.5%) of the infertile group and 44 (14.6%) of the fertile group reported that they had a history of sexually transmitted disease (STD). The mean frequency of coitus per week was 2.90 with ± 0.98 standard deviations (SD) among infertile women and 3.17 with ± 0.94 standard deviations (SD) among fertile women. About 86 (30%) of infertile women and 52 (17.3%) of fertile women reported that they had pain during sexual intercourse or dyspareunia. Table 2 summarizes the obstetrics and sexual characteristics of study participants.

**Table 2:** Obstetrics and sexual characteristics of study participants among infertile and fertile women in public hospitals, Addis Ababa, Ethiopia

Variable	Response	Infertile women (*N* = 287) Frequency(percentage)	Fertile women (*N* = 301) Frequency(percentage)
History of pregnancy	Yes	56 (19.5)	301 (100)
	No	231 (80.5)	0 (0)
Number of children	Mean ± SD	1.0 ± 0.00	2.68 ± 1.2
Previous Number of sexual partner	Mean ± SD	2.51 ± 0.94	2.32 ± 0.8
	< 2	139 (48.4)	188 (62.5)
	≥ 2	148 (51.6)	113 (37.5)
History of STD	Yes	122 (42.5)	44 (14.6)
	No	165 (57.5)	257 (85.4)
Frequency of coitus per week	Mean ± SD	2.9 ± 0.98	3.17 ± 0.94
	< 3	182 (63.4)	181(60.1)
	≥ 3	105 (36.6)	120 (39.9)
Dyspareunia	Yes	86 (30)	52 (17.3)
	No	201 (70)	249 (82.7)

### Past gynaecologic history of the study participants

About 41 (13.6%) of fertile and none of infertile women had a previous history of abortion. The mean number of abortions among those fertile women who had a previous history of abortion was 1.12 with ± 0.33 standard deviation (SD). About 27 (9.4%) and 94 (32.8%) infertile and 71 (23.6%) and 69 (22.9%) of fertile women reported that they had a history of irregular menses and dysmenorrhea or pain during menses, respectively. Regarding previous history of surgery, about 103 (35.9%) of infertile and 31 (10.3%) of fertile women reported that they had a previous history of surgery, and among those who had a previous history of surgery, about 92 (89.3%) of infertile and 14 (45.2%) of fertile women had gynecologic surgery. Regarding the previous history of the gynecologic disease, about 115 (40.1%) of infertile and 26 (12%) of fertile women had a previous history; among those who had a previous history of the disease, about 84 (73%) of infertile and 25 (69.4%) of fertile women had pelvic inflammatory disease (PID). Table 3 shows a summary of the past gynecologic characteristics of study participants.

**Table 3:** Characteristics of past gynecologic history of study participants in public hospitals, Addis Ababa, Ethiopia

Variable	Response	Infertile women (*N* = 287) Frequency(percentage)	Fertile women (*N* = 301) Frequency(percentage)
History of abortion	Yes	0 (0)	41(13.6)
	No	287 (100)	260 (86.4)
Number of abortion	Mean ± SD	0.00	1.12 ± 0.33
History of irregular menses	Yes	27 (9.4)	71(23.6)
	No	260(90.6)	230 (76.4)
Previous history of surgery	Yes	103 (35.9)	31 (10.3)
	No	184 (64.1)	270 (89.7)
Dysmenorrhea	Yes	94(32.8)	69 (22.9)
	No	193(67.2)	232 (77.1)
Types of Previous history of surgery	Abdominal surgery	6 (5.8)	10 (32.3)
	Gynaecologic surgery	92 (89.3)	14 (45.2)
	Others	5 (4.9)	7 (22.6)
Previous history of gynecologic disease	Yes	115 (40.1)	36 (12)
	No	172 (59.9)	265 (88)
Types of previous history of gynecologic disease	Uterine disease	19 (16.5)	8 (22.2)
	Ovarian disease	12 (10.4)	3 (8.3)
	PID	84 (73)	25 (69.4)

### Disease-specific characteristics of study participants

Among those infertile women about 231(80.5%) and 56 (19.5%) had primary and secondary infertility respectively with mean duration of infertility 3.6 years with ± 1.5 standard deviation (SD). All of the infertile women are under treatment, and the mean duration of treatment was 2.93 years with ± 1.32 standard deviation (SD). Table 4 shows a summary of the disease-specific characteristics of study participants.

**Table 4:** Disease-specific characteristics of study participants in public hospitals, Addis Ababa, Ethiopia, November 2022

Variable	Response (*N* = 287)	Frequency	Percent
Duration of infertility (in years)	Mean ± SD	3.6 ± 1.5	
	< 2	72	25.1
	≥ 2	215	74.9
Duration of treatment (in years)	Mean ± SD	2.93 ± 1.32	
	< 2	152	53
	≥ 2	135	47

### Comparison of magnitude of health-related quality of life among study participants

On independent t-test analysis based on transformed score, there was a statistically significant difference between infertile women and fertile women in three of four WHOQOL-BREF domains. The mean transformed score difference between infertile women and fertile women Physical health domain was (t(586) = 1.785, *p* = 0.075,95%CI = -0.069, 1.4605), Psychological Health Domain was (t(586) = -15.510, *p* =  < 0.0001,95%CI = -18.296,-14.183), Social Relationships Domain was (t(586) = -7.465,p =  < 0.0001,95%CI = -20.348,-11.87), Environment Health Domain was (t(586) = 12.350, *p* =  < 0.0001,95%CI = 5.944, 8.239). And the total mean of HRQoL was (t (586) = -8.268, *p* =  < 0.0001, 95%CL = -7.600,-4.6823). Table 5 shows the comparison of the WHOQOL-BREF domain transformed score based on an independent sample t-test. Based on the findings from the study, psychological health, social relations domain, and total mean health-related quality of life are significantly higher in fertile women compared with infertile women. Whereas the environmental domain of health-related quality of life was found to be higher in infertile women compared with fertile women, though not statistically significant, the physical domain of health-related quality of life was found to be higher in infertile women compared with fertile women.

**Table 5:** Summary of independent sample t-test for WHOQOL-BREF domains among fertile and infertile women in public hospitals, Addis Ababa, Ethiopia

WHOQOL‑BREF Domains	Infertile women(*N* = 287) Mean ± SD	Fertile women (*N* = 301) Mean ± SD	t-value	Mean difference	95%CI		*p*-value
					Lower	Upper	
Physical Health Domain	52.45 ± 4.29	51.76 ± 5.14	1.785	0.1947	-0.069	1.4605	0.075
Psychological Health Domain	57.69 ± 14.26	73.93 ± 10.80	-15.510	-16.239	-18.296	-14.183	< 0.0001*
Social Relationships Domain	62.57 ± 29.59	78.68 ± 21.99	-7.465	-16.109	-20.348	-11.87	< 0.0001*
Environment Health Domain	93.45 ± 4.51	86.37 ± 8.82	12.350	7.087	5.944	8.239	< 0.0001*
Total mean HRQoL	66.54 ± 10.18	72.68 ± 7.57	-8.268	-6.14	-7.600	-4.6823	< 0.0001*

^*^Statistically significant = *p* ≤ 0.05, *N* = number of respondents, *SD* Standard deviation

### Factors associated with HRQoL for the infertile population

After checking the assumptions of linear regression analysis (See Annex for the assumptions), both simple and multivariable linear regressions were done to show the significance of each independent variable in predicting the total mean of HRQoL.

The socio-demographic variables, past obstetrics, sexual characteristics, and past Gynecologic characteristics of study participants in public hospitals were analyzed in bivariate analysis. From the socio-demographic variables & clinical characteristics, the following variables were significant at different degrees of p-value ≤ 0.05 for different subdomains of HRQoL as mentioned in Table 14 (See Annex).To identify associated factors for the level of total mean of HRQoL, only those independent variables that were significant at *p* ≤ 0.25 for infertile women were taken from simple linear regression to run multiple linear regressions.

Independent factors such as age, Duration of marriage, Household income, working hours per day, Number of previous sexual partners, Number of coitus per week, History of STD, previous history of pregnancy, Dysmenorrhea, Previous history of surgery, previous history of the gynecologic disease, educational status of infertile women, educational status of the husband of infertile women, Maternal Occupation, types of surgery, types of history of the gynecologic disease, duration of infertility, types of infertility, types of treatment were candidate variables for multiple linear regression analysis in the total mean of HRQoL with *p*-value ≤ 0.25.

After multiple regression results, those independent variables whose VIF (variance inflation factor ≥ 10 were removed starting from the highest one to the lowest in a stepwise manner until the VIF of all independent variables became in acceptable range. Based on colinearity statistics independent variables age, maternal occupation husband occupation, types of gynecologic surgery, previous gynecologic disease, types of surgery, history of gynecologic disease, maternal occupation, and husband occupation were removed from the multiple linear regression, and analysis was done again. The multiple linear regression output showed that duration of the marriage, working hours per day, number of previous sexual partners, types of infertility, and previous history of sexually transmitted disease were found to be statistically significant factors associated with the total mean of HRQoL with, F (17,269) = 45.369, *p* < 0.0001 and Adjusted R^2^ = 0.7252. This implies that approximately 72.5% of the variation in the dependent variable is explained by the independent variable(s) in the model.

For every one standard deviation increase in the duration of marriage, HRQoL was decreased by 0.529 standard deviations, while holding all other variables constant in the model. For every one standard deviation increase in total working hours, HRQoL was decreased by 0.345 standard deviations times, while holding all other variables constant in the model, for every one standard deviation increase in a previous number of sexual partners, HRQoL was decreased by 0.41 standard deviations times, while holding all other variables constant in the model. (β = -0.529, t = -6.53, *p* =  < 0.0001, β = -345, t = -7.6, *p* = 0.001, β = -4.201, t = -3.96, *p* = 0.001).

Infertile women who had no previous history of sexually transmitted disease have 0.277 standard deviation times higher HRQoL score compared with those infertile women who had previous history while holding all other variables constant in the model (β = 0.277, t = 1.667, *p* = 0.035).

Infertile women who had primary types of infertility have a 0.34 standard deviation times less HRQoL score compared with those infertile women who had secondary types of infertility while holding all other variables constant in the model. (β = -0.34, t = -1.667, *p* = 0.049). A summary of multiple linear regressions for the infertile population is given in the following table (Table 6).

**Table 6:** Summary of Multiple Linear Regression with study variables among infertile women in Public hospitals, Addis Ababa, Ethiopia

		**Total Mean HRQoL**				
		**Unstandardized βeta (95% CI)**	**Standardized βeta**	**t-statistics**	**Standard error (SE)**	***P*-value**
Age of respondent		0.398(0.1240,0.673)	0.120	3.614	0.122	0.461
Duration of marriage		-2.545 (-0.305,-1.66)	-0.529	-12.214	0.988	** < 0.0001**
Household income		0.0001(-0.0001,0.0012)	-0.126	1.691	0.0001	0.384
Working hours per day		-1.083 (0.360,1.806)	-0.345	-0.043	0.183	0.001*
Number of previous sexual partners		-6.368 (-7.387,-5.349)	-0.410	-5.559	0.474	0.001*
Number of coitus per week		4.701 (-5.785,-3.617)	0.060	-0.827	0.243	0.218
Duration of treatment		0.17(-0.048,2.662)	0.03	-2.521	0.012	0.059
Previous history of pregnancy	Yes (Ref)					
	No	-3.963 (1.008,6.918)	-0.630	-4.613	0.790	0.080
History of STD	Yes(Ref)					
	No	-6.816 (-4.554,-9.078)	-0.277	-5.344	1.054	0.035*
Dysmenorrhea	Yes (Ref)					
	No	4.867 (4.067,7.328)	0.670	-0.661	0.653	0.088
Previous history of surgery	Yes (Ref)					
	No	0.67 (3.06,5.33)	-0.140	-1.606	1.959	0.109
Previous history of gynecologic disease	Yes (Ref)					
	No	0.07 (-2.37,0.33)	0.017	-2.521	1.830	0.139
Educational status of infertile women	Primary Education (Ref)					
	Secondary Education	-0.108 (-0.08,4.54)	-0.012	0.262	1.498	0.069
	College/ University	-0.016 (-5.21,9.04)	-0.012	3.480	1.165	0.0598
Husband Education	Primary Education (ref)					
	Secondary Education	-0.08(-0.015,8.46)	-0.081	-4.890	1.077	0.052
	College/ University	-0.03(-0.025,4.46)	-0.001	3.046	1.122	0.089
Types of Treatment	Ovulation Induction (Ref)					
	IVF	-0.015 (-1.91,1.31)	-0.039	-0.852	1.363	0.714
	IUI	-1.72 (-8.05,4.612)	0.069	1.260	1.086	0.593
	Surgery	0.018 (-1.56,2.37)	0.022	0.705	2.599	0.685
Types of Surgery	Abdominal surgery (Ref)					
	Gynecologic surgery	-12.035(-16.403,-7.667)	-0.490	-5.412	2.224	0.0001*
	Other surgery	-0.855 (-5.960,4.249)	-0.013	-0.329	2.599	0.742
Types of previous history of gynecologic disease	Uterine disease (Ref)					
	Ovarian disease	-6.951(-11.352,-2.550)	-0.116	-3.102	2.241	0.002
	PID	-2.809 (-6.458,0.840)	-0.166	-1.512	1.858	0.131
Types of Infertility	Primary (ref)					
	Secondary	-1.87 (-2.85,-1.43)	-0.34	-2.11	1.423	0.049*

*B* Unstandardized regression coefficient*, β* Standardized regression coefficient, *statistically significant = *p* ≤ *0.05, ref.* Reference category

## Discussion

The findings of the study indicated that there were significant differences in the magnitude of HRQoL between infertile and fertile women. Fertile women had a higher magnitude of HRQoL in the psychological and social relation domains as well as in the total mean or overall quality of life. On the other hand, infertile women had a higher magnitude of HRQoL in the environmental domain. There was no significant difference in the magnitude of HRQoL in the physical domain between the two groups. Overall, this study highlights the importance of addressing HRQoL issues in infertile women, particularly in the psychological and social relation domains. It also emphasizes the need to consider various factors when designing interventions to improve HRQoL in both fertile and infertile women.

The result of this study showed that there were statistically significant differences in the psychological domain, social relation domain, environmental domain, and total mean of quality of life among infertile women and fertile women, but the difference was not significant in the physical domain. Infertile women were found to have lower quality in the psychological domain, social relation domain, and total mean of overall quality of life, whereas higher in the environmental domain Compared with their fertile counter. The physical domain finding is consistent with the findings of the studies done in Poland, Iran, and Pakistan [25–27]. In contrast to our study, the physical area of the quality of life was significantly different and higher in infertile women as compared with fertile women in the studies done in Arak, Iran, and Nigeria [28, 29]. The lack of difference concerning the physical domain of the quality of life in this study might be attributed to Societal or cultural factors. Since the study participants share common cultural norms or societal expectations regarding physical health, it could contribute to the observed similarity in HRQoL scores in the physical domain.

Regarding the psychological and social relation domain, the present study result showed that the psychological and social relation domain of health-related quality of life is lower among infertile women compared with fertile women and the difference was statistically significant. This finding is consistent with similar studies done in Iran, Poland, Arak, Pakistan, India, and California [2, 25, 26, 30, 31]. In contrast to the study findings from Nigeria [1]. Study findings in the psychological and social relation domain can be explained by the fact that infertile Women face serious emotional turmoil due to infertility and Emotions can range from confusion, anger, hopelessness, feelings of unworthiness, and frustration, to denial, withdrawal, social isolation and depression [2]. The study findings from the Nigerian study in the psychological and social relation domain might be attributed to sociocultural differences among the study participants.

On the other hand, the environmental domain of quality of life was found to be significantly different and higher among the infertile group compared with the fertile group. This finding is in line with similar study results from Nigeria, Arak, and India [1, 25, 31]. This might be explained by the fact that most of my study participants are in the early phase of infertility treatment, which might increase the feeling of safety increasing the environmental domain. And in contrast with a study finding from Iran [2]. The difference in my study findings from the Iranian study might be attributed to the differences in environmental factors and socio-economic.

Regarding the total overall mean of HRQoL, my study showed that the overall mean quality of life between infertile and fertile women has a statistically significant difference. This finding is consistent with a similar study from India and China [31, 32]. And in contrast with findings from a similar Nigerian study. In explaining the difference in the total mean quality of life of infertile women and fertile women, it can be said that the quality of life in infertile women is related to the amount of pressure of people around to give birth, the intensity of desire for having a child, an individual’s assessment of the household’s economic situation and irrational thoughts related to having a child and the duration and cause of infertility. Infertile women also suffer more stress and with increasing stress, their therapeutic response decreases and leads to a decline in quality of life. Attitudes toward the issue of women’s infertility are often affected by racial and cultural differences, and on this basis, culture affects the quality of life of infertile people [14].

In this study, women’s previous number of sexual partners has a statistically significant association with total mean HRQoL which was consistent with studies done in Iran and Poland [2, 30]. This might be explained by the fact that most women with infertility think that given the increased number of sexual partners, they hadn’t given birth leading them to think that the problem might be with them. Also having a history of an increased number of sexual partners is a social taboo that might have negatively impacted quality of life [15].

On the other hand duration of marriage was found to be negatively associated with the total mean of HRQoL. This finding is consistent with findings from similar studies done in Poland, India, and Pakistan [3, 30, 26]. This might be due to the reason that as the duration of marriage increases, then the community, family, and oneself expect a couple to have a child so failing to have a child as the duration of marriage increases results in putting social pressure and stress to the couple leading to affect the quality of life negatively.

In the present study, having a previous history of sexually transmitted disease is significantly associated with total mean HRQoL. This finding is similar to another study done in India, Pakistan, and Poland [30, 28, 33]. This can be explained by the fact that having a sexually transmitted disease is one of the well-identified factors for having infertility problems which has been told to patients with STDs and infertility so that clients might think that having the disease might have caused the infertility problem [34].

On the other hand total number of working hours was also found to be negatively associated with the total mean HRQoL in my study. This finding is in line with study findings from Korea [35]. This might be because working long hours can affect the relationship between a husband and a wife with less time for conversation and a sexual life. This could result in negative emotions and depression and even affect the plan to have coital frequency for trial of having babies leading to affect quality of life.

The study also showed types of infertility were found to be associated with total mean HRQoL. Infertile women having primary type infertility have lesser total mean HRQoL compared with infertile women who have secondary type of infertility. This finding is in line with similar study findings from Egypt [16]. This might be because women with secondary types of infertility had at least one successful previous pregnancy boosts their psychology and enables them to think that they have a better chance of conceiving another child compared with those infertile women who had primary types of infertility.

### Strengths and limitations of the study

As a strength, the study was conducted in such a way that it involved all public hospitals where infertility treatment is being given by subspecialists to maximize the representativeness of the study. Both the domains and the summary measures were reported which will help to avoid the information loss that could have occurred in reporting only the summary measures. The other strength of this study includes the fact that it was the first study to examine and compare the quality of life between infertile and fertile women in Ethiopia and East Africa, and internationally and nationally validated questionnaires on quality of life provide great insight for further study to be conducted in the area. This study is not without limitations which do not necessarily invalidate the findings from this study. One of the limitations is the tool used doesn’t assess disease-specific quality of life, so disease-related quality of life couldn’t be assessed. The study was hospital-based rather than a community study and it is expected that women attending the clinic may have social support from their spouses and relatives. A community-based study might reveal different findings concerning QoL among infertile women who have decided to go through other treatment options or not to undergo any treatment at all. The study would also have been better studied in couples than in women alone. The other limitation of my study was only public hospitals were included, private infertility centers were not considered except for pretesting the questionnaire.

## Conclusions

In conclusion, the study found that there is a significant difference in the magnitude of health-related quality of life among infertile women and fertile women in terms of psychological, social relation, environmental domains, and overall quality of life. Fertile women had a higher magnitude of health-related quality of life in terms of psychological and social relation domains, while infertile women had a higher magnitude of health-related quality of life in the environmental domain. The study also revealed that several factors were found to be statistically significant in affecting the total mean of HRQoL for both fertile and infertile women, including the age of the woman, number of previous sexual partners, and history of pregnancy or previous gynecologic disease. For infertile women specifically, factors such as duration of marriage, working hours per day, types of infertility, and previous history of sexually transmitted disease were also significant. For fertile women, factors such as the educational level of the husband and maternal and husband's occupation were significant.

Based on the study findings. It is recommended that the Ministry of Health and researchers develop multidimensional interventions that aim at empowering infertile women by targeting their psychological, social, and environmental needs. Researchers could explore interventions that could be implemented to improve the health-related quality of life of infertile women, taking into consideration the impact of various social, psychological, and environmental factors. Given the significant impact on quality of life, addressing the psychological needs of infertile women becomes crucial. Support groups, therapy, and stress management techniques can be powerful tools, equipping them to cope with the emotional challenges of infertility and navigate the often challenging path to parenthood.

### Supplementary Information

Supplementary Material 1.

## Data Availability

The data underlying this article will be shared on reasonable request to the corresponding author**.**

## Abbreviation

QoL: Quality of life
GMH: Gandhi Memorial Hospital
HRQoL: Health Related Quality of Life
IUI: Intrauterine insemination
IVF: Invitro fertilization
SPHMMC: St. Paul’s Hospital Millennium Medical College
STD: Sexually transmitted disease
TASH: Tikur Anbessa Specialized Hospital
WHO: World Health Organization
WHOQOL-BREF: Abbreviated World Health Organization Quality of Life questionnaire
