# Supplementary material for: Health-related quality of life and its associated factors among infertile women compared with fertile women in public hospital Addis Ababa, Ethiopia: a comparative cross-sectional study
Source: BMC Womens Health. 2024 Jul 23;24:417. doi: 10.1186/s12905-024-03163-3 (PMC11264358; doi:10.1186/s12905-024-03163-3)
Supplement: Supplementary file 1 — Supplementary Material 1. [file 12905_2024_3163_MOESM1_ESM.docx]

**Supplementary File**

## Table S1: Summary of simple Linear Regression with study variables among infertile women in public hospitals, Addis Ababa

|  |  | **Physical Domain** | | **Psychological Domain** | | **Social Relation Domain** | | **Environmental Domain** | | **Total mean of HRQoL** | |
| --- | --- | --- | --- | --- | --- | --- | --- | --- | --- | --- | --- |
|  |  | **β (95% CI)** | **P-value** | **β (95% CI)** | **P-value** | **β (95% CI)** | **P-value** | **β (95% CI)** | **P-value** | **β (95% CI)** | **P-value** |
| Age |  | 0.110  (-0.029,0.249) | 0.121 | -0.62  (-0.391,0.267) | 0.711 | -2.394  (-3.315,-1.47) | 0.001* | -0.086  (-0.232,0.61) | 0.250 | -0.993  (-1.304,-0.683) | <0.001* |
| Place of residence | Rural(Ref) |  |  |  |  |  |  |  |  |  |  |
|  | Urban | 4.423  (2.284,6.563) | 0.0001* | -0.664  (-9.311,7.982) | 0.880 | 4.408  (-13.52,22.34) | 0.629 | -0.784  (-4.557,2.989) | 0.683 | 1.497  (-4.674,7.668) | 0.633 |
| Duration of marriage | | 0.210  (-0.007,0.426) | 0.058 | -3.465  (-4.067,-2.862) | <0.0001* | -6.549  (-7.845,-5.25) | <0.0001* | -0.374  (-0.599,-0.149) | 0.001* | -2.545  (-2.969,-2.120) | <0.001* |
| Household income | | -0.088  (-0.001,0.001) | 0.138 | -0.003  (-0.004,-0.002) | <0.0001* | -0.003  (-0.005,0.001) | 0.004* | 0.001  (0.0001,0.001) | 0.002* | -0.001  (-0.002,-0.001) | <0.0001* |
| Working hours per day | | -0.023  (-0.331,0.286) | 0.886 | 0.088  (-0.940,1.115) | 0.866 | 4.581  (2.518,6.644) | <0.0001* | -0.315  (-0.637,0.008) | 0.056 | 1.083  (0.360,1.806) | 0.003* |
| Number of previous sexual partner | | -0.9  (-1.420,-0.380) | 0.001* | -9.139  (-10.547,7.731) | <0.001* | -14.544  (17.79,11.29) | 0.001* | -0.889  (-1.437,-0.341) | 0.002* | -6.368  (-7.387,-5.349) | <0.0001* |
| Number of coitus per week | | -0.546  (-1.053,-0.039) | 0.035* | -6.241  (-7.779,-4.703) | <0.0001* | -11.520  (-14.78,8.256) | <0.0001* | -0.499  (-1.033,0.035) | 0.067 | -4.701  (-5.785,-3.617) | <0.0001* |
| Previous history of pregnancy. | Yes (Ref) |  |  |  |  |  |  |  |  |  |  |
|  | No | -0.758  (-2.013,0.498) | 0.236 | 8.635  (4.569,12.702) | <0.0001* | 4.527  (-4.147,13.20) | 0.305 | 3.448  (2.187,4.709) | <0.0001* | 3.963  (1.008,6.918) | 0.009* |
| History of STD | Yes(Ref) |  |  |  |  |  |  |  |  |  |  |
|  | No | -1.032  (-2.033,-0.030) | 0.044* | 10.414  (7.283,13.544) | <0.0001* | 15.809  (9.09,22.527) | <0.0001* | 2.073  (1.04,3.106) | <0.0001* | 6.816  (4.554,9.078) | <0.0001* |
| Dysmenorrhea | Yes (Ref) |  |  |  |  |  |  |  |  |  |  |
|  | No | 0.999  (-0.066,2.047) | 0.066 | 10.058  (6.721,13.395) | <0.0001* | 8.413  (1.141,15.68) | 0.024* | 0.008  (-1.110,1.125) | 0.989 | 4.867  (4.06,7.328) | <0.0001* |
| Oligomenorrhea | Yes (Ref) |  |  |  |  |  |  |  |  |  |  |
|  | No | -1.966  (-3.659,-0.273) | 0.023* | 12.923  (7.439,18.406) | <0.0001* | -11.674  (-23.392,0.04) | 0.051 | -0.325  (-2.12,1.471) | 0.722 | -0.261  (-4.32,3.799) | 0.900 |
| Types of infertility | Primary (Ref) |  |  |  |  |  |  |  |  |  |  |
|  | Secondary | 0.758  (-0.498,2.013) | 0.236 | -8.635  (-12.702,4.569) | <0.0001* | -4.527  (-13.20,4.147) | 0.305 | -3.448  (-4.709,-2.187) | <0.0001* | -3.963  (-6.918,-1.008) | 0.009* |
| Duration of treatment | | 0.355  (-0.021,0.730) | 0.064 | -0.487  (-6.35,-4.155) | <0.0001* | -9.036  (-11.42,6.651) | <0.001* | 0.126  (-0.271,0.522) | 0.534 | -3.452  (-4.254,-2.650) | <0.0001* |
| Previous history of surgery | Yes (Ref) |  |  |  |  |  |  |  |  |  |  |
|  | No | -0.936  (-1.970,0.098) | 0.076 | 15.472  (12.519,18.426) | <0.0001* | 34.880  (28.96,40.79) | <0.0001* | -0.222  (-1.315,0.871) | 0.690 | 12.299  (10.286,14.311) | <0.0001* |
| previous history of gynecologic disease | Yes (Ref) |  |  |  |  |  |  |  |  |  |  |
|  | No | -1.091  (-2.101,-0.082) | 0.034* | 13.021  (9.992,16.049) | <0.0001* | 33.675  (27.85,39.53) | <0.0001* | -0.808  (-1.874,0.258) | 0.337 | 11.199  (9.163,13.235) | <0.0001* |
| Educational status of infertile women | Primary Education (Ref) |  |  |  |  |  |  |  |  |  |  |
|  | Secondary Education | -1.012  (-2.786,0.763) | 0.263 | -5.204  (-8.894,-1.515) | 0.006* | -12.416  (-19.89,-4.94) | 0.0001* | 4.372  (1.359,7.384) | 0.005* | -3.565  (-6.153,-0.977) | 0.007* |
|  | College/  University | 1.788  (0.546,3.029) | 0.005* | -7.16  (-11.261, 3.05) | 0.001* | -41.504  (-48.72,34.28) | 0.0001* | -0.952  (-2.271,0.365) | 0.156 | -11.956  (-14.602,-9.310) | 0.0001* |
| Educational status of husband of infertile women | Primary Education (Ref) |  |  |  |  |  |  |  |  |  |  |
|  | Secondary Education | -0.475  (-3.586,2.6377) | 0.764 | -6.364  (-12.86,0.135) | 0.055 | -23.48  (-36.52,10.43) | 0.0001* | -0.3  (-5.64,5.04) | 0.912 | -7.654  (-12.155,-3.154) | 0.0001* |
|  | College/  University | -2.549  (-5.13,0.0323) | 0.053 | 0.664  (-7.98,9.311) | 0.151 | -4.408  (22.34,13.52) | 0.629 | 0.306  (-2.425,3.037) | 0.826 | -1.497  (-7.668,4.674) | 0.633 |
| Maternal Occupation | Private employee (Ref) |  |  |  |  |  |  |  |  |  |  |
|  | House wife | 1.362  (-0.181,2.906) | 0.083 | -10.27  (-15.40,-5.138) | <0.0001* | -28.446  (-38.79,18.10) | <0.0001* | 2.89  (1.256,4.533) | 0.001* | -8.615  (-12.255,-4.974) | <0.0001* |
|  | Government employee | -0.796  (-2.283,0.69) | 0.292 | -10.339  (-15.28,-5.398) | <0.0001* | -11.710  (-21.67,-1.75) | 0.021* | 2.432  (0.855,4.009) | 0.003* | -5.103  (-8.608,-1.599) | 0.004* |
|  | NGO | -4.167  (-10.13,1.793) | 0.170 | -4.06  (-23.87,15.755) | 0.687 | -24.679  (-64.61,15.25) | 0.225 | 2.564  (-3.761,8.89) | 0.426 | -7.585  (-21.64,6.47) | 0.289 |
| Husband occupation of infertile group | Private employee (Ref) |  |  |  |  |  |  |  |  |  |  |
|  | Daily laborer | -0.982  (-3.164,1.199) | 0.376 | -9.063  9-16.16,-1.957) | 0.013* | -43.33  (60.37,81.3) | ,0.0001* | -0.625  (-2.91,1.66) | 0.59 | -13.5  (-18.29,-8.71) | 0.0001* |
|  | Government employee | -1.277  (-3.092,0.539) | 0.167 | -8.147  9-14.06,-2.232) | 0.007* | -3.073  (-14.09,7.94) | 0.583 | -0.829  (-2.73,1.07) | 0.391 | -3.33  (-7.32,0.656) | 0.101 |
|  | NGO | -0.00019  (-6.218,6.218) | 1.000 | 18.229  (-2.025,38.484) | 0.078 | 20.83  (-16.89,58.56) | 0.278 | 5.859  (-0.644,12.36) | 0.077 | 11.23  (-2.424,24.89) | 0.107 |
| Types of infertility treatment | Ovulation induction (Ref) |  |  |  |  |  |  |  |  |  |  |
|  | IVF | 0.805  (-0.621,2.23) | 0.267 | -3.638  (-8.372,1.096) | 0.132 | -10.796  (-20.61,-0.98) | 0.031* | -1.461  (-2.952,0.03) | 0.055 | -3.773  (-7.143,-0.402) | 0.028* |
|  | IUI | -0.21  (-2.116,1.696) | 0.829 | -1.72  (-8.052,4.612) | 0.593 | -5.335  (18.46,7.789) | 0.424 | -0.787  (-2.781,1.207) | 0.438 | -2.013  (-6.521,2.495) | 0.380 |
|  | Surgery | -0.163  (-1.714,1.387) | 0.836 | -5.256  (-10.41,-0.106) | 0.045* | -9.122  (-19.795,1.55) | 0.094 | -1.951  (-3.572,-0.329) | 0.019* | -4.123  (-7.789,-0.457) | 0.028* |
| Types of surgery | Abdominal surgery(Ref) |  |  |  |  |  |  |  |  |  |  |
|  | Gynecologic surgery | 1.367  (0.307,2.427) | 0.012* | -16.579  (-19.59,-13.57) | <0.0001* | -36.518  (-42.59,30.44) | <0.0001* | 0.192  (-0.938,1.322) | 0.738 | -12.885  (-14.949,-10.82) | <0.0001* |
|  | Other surgery | -2.049  (-5.83,1.732) | 0.287 | -4.759  (-15.492,5.975) | 0.384 | -16.228  (37.895,5.44) | 0.142 | 0.362  (-3.669,4.393) | 0.177 | -5.668  (-13.03,1.694) | <0.0001* |
| Types of previous history of gynecologic disease | Uterine disease (Ref) |  |  |  |  |  |  |  |  |  |  |
|  | Ovarian disease | 2.295  (-0.206,4.797) | 0.072 | -9.637  (-16.853,-2.42) | 0.009* | -34.188  (-35.39,-21.9) | <0.0001* | -0.014  (-2.655,2.628) | 0.992 | -10.386  (-15.374,-5.19) | <0.0001* |
|  | PID | 0.637  (-0.463,1.737) | 0.255 | -15.936  (-19.11,-12.76) | <0.0001* | -28.632  (-35.39,21.88) | <0.0001* | 0.73  (-0.431,1.892) | 0.217 | -10.8  (-13.08,-8.518) | <0.0001* |

*B= Unstandardized regression coefficient, β = Standardized regression coefficient, *statistically significant= p≤ 0.05, ref. = Reference category*

| Variables | | N | Mean ± SD | t-value | P-value | 95%CI | | Mean differences |
| --- | --- | --- | --- | --- | --- | --- | --- | --- |
|  |  |  |  |  |  | Lower | Upper |  |
| Place of residence | Urban | 276 | 66.60 ±10.38 | 2.395 | 0.633 | -4.6744 | 7.668 | 1.4968 |
|  | Rural | 11 | 65.10 ±10.38 |  |  |  |  |  |
| Age of respondent in years | <35 | 243 | 67.38 ± 10.08 | 3.314 | 0.001* | 2.206 | 8.663 | 5.43492 |
|  | ≥ 35 | 44 | 61.94 ± 9.49 |  |  |  |  |  |
| Duration of marriage in years | < 5 | 198 | 69.08 ± 9.63 | 6.758 | <0.0001* | 5.78951 | 10.5475 | 8.1685 |
|  | ≥ 5 | 89 | 60.91 ± 9.11 |  |  |  |  |  |
| Age at marriage in years | <25 | 136 | 67.74 ± 9.80 | 1.899 | 0.059 | -0.0828 | 4.6349 | 2.27606 |
|  | ≥ 25 | 151 | 65.46 ± 10.46 |  |  |  |  |  |
| Working hours per day | <8 | 240 | 66.49 ± 10.87 | -0.291 | 0.848 | -2.4381 | 1.81256 | -0.31275 |
|  | ≥ 8 | 47 | 66.81 ± 5.58 |  |  |  |  |  |
| Number of previous sexual partners | <2 | 139 | 71.08 ± 11.26 | 7.98 | <0.0001* | 6.62641 | 10.9728 | 8.79963 |
|  | ≥ 2 | 148 | 62.28 ± 6.71 |  |  |  |  |  |
| Number of coitus | <3 | 182 | 70.16 ± 10.04 | 9.851 | <0.0001* | 7.90180 | 11.8486 | 9.8752 |
|  | ≥ 3 | 105 | 60.28 ± 6.88 |  |  |  |  |  |
| Duration of infertility in years | <2 | 72 | 76.46 ± 6.38 | 13.634 | <0.0001* | 11.3204 | 15.153 | 13.2367 |
|  | ≥ 2 | 215 | 63.22 ± 8.99 |  |  |  |  |  |
| Duration of treatment in years | <2 | 152 | 69.25 ± 9.05 | 4.926 | <0.0001* | 3.45303 | 8.052 | 5.75252 |
|  | ≥ 2 | 135 | 63.49 ± 10.55 |  |  |  |  |  |
| Previous history of pregnancy | Yes | 56 | 63.35 ± 9.19 | -2.64 | 0.009* | -6.9183 | -1.0084 | -3.96332 |
|  | No | 231 | 67.32 ± 10.28 |  |  |  |  |  |
| Previous history of STD | Yes | 122 | 62.63 ± 7.78 | -6.219 | <0.0001* | -8.9732 | -4.6589 | -6.81604 |
|  | No | 165 | 69.44 ± 10.79 |  |  |  |  |  |
| Dyspareunia | Yes | 86 | 68.28 ± 10.32 | -1.896 | 0.059 | -0.0941 | 5.04826 | 2.47709 |
|  | No | 201 | 65.80 ± 10.06 |  |  |  |  |  |
| Oligomenorrhea | Yes | 27 | 66.78 ± 1.42 | 0.363 | 0.717 | -1.1508 | 1.67172 | 2.6051 |
|  | No | 260 | 66.52 ± 10.69 |  |  |  |  |  |
| Dysmenorrhea | Yes | 94 | 63.27 ± 10.35 | -3.812 | <0.0001* | -7.3873 | -2.3472 | -4.86723 |
|  | No | 193 | 68.14 ± 9.74 |  |  |  |  |  |

# **Table S2**: Mean Quality of life difference among independent variables

**Table S3**: Summary of One way ANOVA with study variables among infertile groups in public hospital, Addis Ababa, Ethiopia

| **Variables** | | **Mean±SD** | **Test of Homogeneity of variances** | | **ANOVA** | | **Group differences** | | | | |
| --- | --- | --- | --- | --- | --- | --- | --- | --- | --- | --- | --- |
|  |  |  | **Levene Statistic** | **P-value** | **F** | **P-value** |  | **Mean difference** | **P-value** | **95% CI** | |
|  |  |  |  |  |  |  |  |  |  | **Lower** | **Upper** |
| Educational status of infertile women | Primary education | 59.92 ±5.70 | 13.135 | <0.0001* | 44.91 | <0.0001* | Primary –secondary | -10.07 | <0.0001* | -13.0866 | -7.0573 |
|  | Secondary education | 66.99 ±10.15 |  |  |  |  | Primary –college | -13.5265 | <0.0001* | -16.189 | -10.864 |
|  | College/university | 70.45 ± 8.96 |  |  |  |  | Secondary -college | -3.4545 | 0.021* | -6.5152 | -0.3939 |
| Educational status of husband of infertile women | Primary education | 65.10 ± 000 | 9.475 | <0.0001* | 15.749 | <0.0001* | Primary –secondary | 4.20879 | 0.003* | 1.2196 | 7.1979 |
|  | Secondary education | 60.89 ± 10.07 |  |  |  |  | Primary –college | -3.36217 | <0.0001* | -4.9982 | -1.7262 |
|  | College/university | 68.47 ± 9.80 |  |  |  |  | Secondary -college | 7.57096 | <0.0001* | 4.1834 | 10.9585 |
| Maternal Occupation | Private employee | 72.24 ± 7.37 | 7.815 | <0.0001* | 7.588 | <0.0001* | Private employee-House wife | 8.6142 | <.0001* | 4.2877 | 12.9416 |
|  | House wife | 63.63 ± 11.23 |  |  |  |  | Private employee-Governmental employee | 5.10339 | 0.003* | 1.2762 | 8.9306 |
|  | Government employee | 67.14 ± 9.36 |  |  |  |  | Private employee-NGO | 7.58547 | 0.612 | -70.4973 | 85.6683 |
|  | NGO | 64.66 ± 5.84 |  |  |  |  | House wife -NGO | -1.02915 | 1.000 | -82.1297 | 80.0714 |
| Husband occupation of infertile group | Private employee | 70.91 ± 8.89 | 3.036 | 0.03* | 16.953 | <0.0001* | Private employee-Daily laborer | 13.50 | <0.0001* | 7.6920 | 19.3095 |
|  | Daily laborer | 57.41 ± 6.71 |  |  |  |  | Private Employee-Governmental Employee | 3.33 | 0.433 | -2.0935 | 8.7565 |
|  | Government employee | 67.58 ± 9.90 |  |  |  |  | Private Employee-NGO | -11.23 | <0.0001* | -16.4195 | -6.0411 |
|  | NGO | 82.14 ± 0.00 |  |  |  |  | Daily laborer -NGO | -24.73 | <0.0001* | -27.663 | -21.799 |
| Types of infertility treatment | Ovulation induction | 69.58 ± 9.72 | 0.448 | 0.719 | 2.074 | 0.10 |  |  |  |  |  |
|  | IVF | 65.80 ± 10.33 |  |  |  |  |  |  |  |  |  |
|  | IUI | 67.56 ± 9.15 |  |  |  |  |  |  |  |  |  |
|  | Surgery | 65.45 ± 10.41 |  |  |  |  |  |  |  |  |  |
| Types of previous history of surgery | Abdominal surgery | 65.10 ± 0.00 | 32.046 | 0.001* | 6.025 | 0.003* | Abdominal surgery-Gynecologic surgery | 7.21605 | <0.0001* | 5.4850 | 8.9471 |
|  | Gynecologic surgery | 57.88 ± 6.83 |  |  |  |  | Abdominal surgery –Other surgery | 0.000 | 1.000 | 0.000 | 0.000 |
|  | Other surgery | 65.10 ± 0.00 |  |  |  |  | Gynecologic surgery –Other surgery | -7.21605 | <0.0001* | -8.9471 | -5.4850 |
| Types of previous history of gynecologic disease | Uterine disease | 62.06 ± 1.85 | 37.10 | <0.0001* | 1.392 | 0.253 |  |  |  |  |  |
|  | Ovarian disease | 59.75 ± 6.67 |  |  |  |  |  |  |  |  |  |
|  | PID | 59.34 ± 7.01 |  |  |  |  |  |  |  |  |  |
| Total household Income | Low | 65.10 ± 0.00 | 14.407 | <0.0001* | 6.568 | 0.002* | Low-Middle | -2.4247 | 0.001* | -3.9581 | -0.8913 |
|  | Middle | 67.53 ± 9.73 |  |  |  |  | Low-High | 3.53077 | 0.185 | -1.1301 | 8.192 |
|  | High | 61.53 ± 12.3 |  |  |  |  | Middle-High | 5.95547 | 0.012* | 1.0702 | 10.8407 |

## Table S4: Summary of Post hoc power analysis for the Health related quality of life differences between the two groups in public hospital, Addis Ababa, Ethiopia

|  | | **Physical**  **Domain** | **Psychological**  **Domain** | **Social Relation domain** | **Environmental**  **Domain** | **Overall mean**  **score** | **Regression** |
| --- | --- | --- | --- | --- | --- | --- | --- |
| **Statistical test** | | Score difference | Score  difference | Score  difference | Score  difference | Score difference | Multiple regression |
| **Input** | Tails | Two | Two | Two | Two | Two | Two |
|  | Effect size ( d) | 0.2513669 | 1.2839096 | 0.6179884 | 1.0107455 | 0.6844709 | 2.636363 |
|  | α err Prob | 0.05 | 0.05 | 0.05 | 0.05 | 0.05 | 0.05 |
|  | Sample size group 1 | 287 | 287 | 287 | 287 | 287 | 287 |
|  | Sample size group 2 | 301 | 301 | 301 | 301 | 301 | 301 |
| **Out put** | Non-centrality parameter | 3.0467977 | 15.5621637 | 7.4905871 | 12.2511639 | 8.2964160 | 756.6364 |
|  | Critical t | 1.9640205 | 1.9640205 | 1.9640205 | 1.9640205 | 1.9640205 | 2.246129 |
|  | Df | 586 | 586 | 586 | 586 | 586 | 281 |
|  | Power (1-β) | 0.8603377 | 1.000000 | 1.000000 | 1.000000 | 1.000000 | 1.0000 |

**Table S5**: Summary of simple linear regression on Total mean HRQoL for the Overall population in public hospital, Addis Ababa, Ethiopia

|  |  | **Total Mean HRQoL** | | | | |
| --- | --- | --- | --- | --- | --- | --- |
|  |  | **Unstandardized βeta**  **(95% CI)** | **Standardized βeta** | **t-statistics** | **Standard error (SE)** | **P-value** |
| Age |  | -0.656 (-0.858,-0.454) | -0.255 | -6.381 | 0.103 | <0.0001 |
| Place of residence | Rural(Ref) |  |  |  |  |  |
|  | Urban | -1.000(-4.282,2.279) | -0.025 | -0.600 | 1.670 | 0.5490 |
| Duration of marriage | | -0.683 (-0.941,-0.426) | -0.211 | -5.214 | 0.131 | <0.0001 |
| Household income | | 0.001 (0.0001,0.0012) | 0.131 | 3.196 | 0.0001 | 0.001 |
| Working hours per day | | 1.092 (0.679,1.504) | 0.210 | 5.199 | 0.210 | <0.0001 |
| Number of previous sexual partner | | -5.194 (-5.963,-4.426) | -0.481 | -13.271 | 0.391 | <0.0001 |
| Number of coitus per week | | -1.107 (-1.896,-0.318) | -0.318 | -2.756 | 0.402 | 0.006 |
| Previous history of pregnancy. | Yes (Ref) |  |  |  |  |  |
|  | No | -3.9.4(-5.440,-2.369) | -0.202 | -4.993 | 0.782 | <0.0001 |
| History of STD | Yes(Ref) |  |  |  |  |  |
|  | No | 8.578(7.025,10.130) | 0.409 | 10.851 | 0.791 | <0.0001 |
| Dysmenorrhea | Yes (Ref) |  |  |  |  |  |
|  | No | 4.597 (2.927,6.267) | 0.218 | 5.407 | 0.850 | <0.0001 |
| Oligomenorrhea | Yes (Ref) |  |  |  |  |  |
|  | No | -1.073 (-0.980,3.126) | 0.042 | 1.026 | 1.045 | 0.305 |
| Previous history of surgery | Yes (Ref) |  |  |  |  |  |
|  | No | 10.366 (8.746,11.987) | 0.461 | 12.563 | 0.825 | <0.0001 |
| previous history of gynecologic disease | Yes (Ref) |  |  |  |  |  |
|  | No | 11.179(9.679,12.680) | 0.517 | 14.636 | 0.764 | <0.0001 |
| Educational status of infertile women | Primary Education (Ref) |  |  |  |  |  |
|  | Secondary Education | -1.819 (-4.867,1.229) | -0.047 | -1.172 | 1.552 | 0.242 |
|  | College/ University | -6.617(-8.355,-4.879) | -0.299 | -7.479 | 0.885 | 0.0001 |
| Educational status of husband of infertile women | Primary Education (Ref) |  |  |  |  |  |
|  | Secondary Education | -4.506 (-10.155,1.142) | -0.065 | -1.567 | 2.876 | 0.118 |
|  | College/University | -0.732 (-3.924,2.460) | -0.019 | -0.450 | 1.625 | 0.650 |
| Maternal Occupation | Private employee (Ref) |  |  |  |  |  |
|  | House wife | -5.772 (-7.880,-3.665) | -0.301 | -5.379 | 1.073 | <0.0001 |
|  | Government employee | -2.988 (-5.113,-0.862) | -0.155 | -2.761 | 1.082 | 0.006 |
|  | NGO | -5.671(-13.967,2.626) | -0.055 | -1.342 | 4.224 | 0.180 |
| Husband occupation of infertile group | Private employee (Ref) |  |  |  |  |  |
|  | Daily laborer | -7.607 (-10.297,-4.917) | -0.256 | -5.554 | 1.370 | 0.0001 |
|  | Government employee | -0.705 (-1.119,2.530) | 0.035 | 0.759 | 0.929 | 0.448 |
|  | NGO | 7.623 (-1.136,14.109) | 0.094 | 2.308 | 3.303 | 0.021 |
| Types of surgery | Abdominal surgery (Ref) |  |  |  |  |  |
|  | Gynecologic surgery | -12.435(-14.159,-10.711) | -0.506 | -14.163 | 0.878 | <0.0001 |
|  | Other surgery | -1.140 (-5.829,3.547) | -0.017 | -0.471 | 2.387 | 0.633 |
| Types of previous history of gynecologic disease | Uterine disease (Ref) |  |  |  |  |  |
|  | Ovarian disease | -13.080 (-17.374,-8.785) | -0.218 | -5.982 | 2.187 | <0.0001 |
|  | PID | -10.625 (-12.368,-8.883) | -0.437 | -11.976 | 0.887 | <0.0001* |

*B= Unstandardized regression coefficient, β = Standardized regression coefficient, *statistically significant= p≤ 0.05, ref. = Reference category*

**Table S6**: Summary of simple linear regression on Total means HRQoL for the fertile population in public hospital, Addis Ababa, Ethiopia

|  |  | **Total Mean HRQoL** | | | | |
| --- | --- | --- | --- | --- | --- | --- |
|  |  | **Unstandardized βeta**  **(95% CI)** | **Standardized βeta** | **t-statistics** | **Standard error (SE)** | **P-value** |
| Age |  | -0.656 (-0.858,-0.454) | -0.255 | -6.381 | 0.103 | <0.0001 |
| Place of residence | Rural(Ref) |  |  |  |  |  |
|  | Urban | -1.000(-4.282,2.279) | -0.025 | -0.600 | 1.670 | 0.5490 |
| Duration of marriage | | -0.683 (-0.941,-0.426) | -0.211 | -5.214 | 0.131 | <0.0001 |
| Household income | | 0.001 (0.0001,0.0012) | 0.131 | 3.196 | 0.0001 | 0.001 |
| Working hours per day | | 1.092 (0.679,1.504) | 0.210 | 5.199 | 0.210 | <0.0001 |
| Number of previous sexual partner | | -5.194 (-5.963,-4.426) | -0.481 | -13.271 | 0.391 | <0.0001 |
| Number of coitus per week | | -1.107 (-1.896,-0.318) | -0.318 | -2.756 | 0.402 | 0.006 |
| Previous history of pregnancy. | Yes (Ref) |  |  |  |  |  |
|  | No | -3.9.4(-5.440,-2.369) | -0.202 | -4.993 | 0.782 | <0.0001 |
| History of STD | Yes(Ref) |  |  |  |  |  |
|  | No | 8.578(7.025,10.130) | 0.409 | 10.851 | 0.791 | <0.0001 |
| Dysmenorrhea | Yes (Ref) |  |  |  |  |  |
|  | No | 4.597 (2.927,6.267) | 0.218 | 5.407 | 0.850 | <0.0001 |
| Oligomenorrhea | Yes (Ref) |  |  |  |  |  |
|  | No | -1.073 (-0.980,3.126) | 0.042 | 1.026 | 1.045 | 0.305 |
| Previous history of surgery | Yes (Ref) |  |  |  |  |  |
|  | No | 10.366 (8.746,11.987) | 0.461 | 12.563 | 0.825 | <0.0001 |
| previous history of gynecologic disease | Yes (Ref) |  |  |  |  |  |
|  | No | 11.179(9.679,12.680) | 0.517 | 14.636 | 0.764 | <0.0001 |
| Educational status of infertile women | Primary Education (Ref) |  |  |  |  |  |
|  | Secondary Education | -1.819 (-4.867,1.229) | -0.047 | -1.172 | 1.552 | 0.242 |
|  | College/ University | -6.617(-8.355,-4.879) | -0.299 | -7.479 | 0.885 | 0.0001 |
| Educational status of husband of infertile women | Primary Education (Ref) |  |  |  |  |  |
|  | Secondary Education | -4.506 (-10.155,1.142) | -0.065 | -1.567 | 2.876 | 0.118 |
|  | College/University | -0.732 (-3.924,2.460) | -0.019 | -0.450 | 1.625 | 0.650 |
| Maternal Occupation | Private employee (Ref) |  |  |  |  |  |
|  | House wife | -5.772 (-7.880,-3.665) | -0.301 | -5.379 | 1.073 | <0.0001 |
|  | Government employee | -2.988 (-5.113,-0.862) | -0.155 | -2.761 | 1.082 | 0.006 |
|  | NGO | -5.671(-13.967,2.626) | -0.055 | -1.342 | 4.224 | 0.180 |
| Husband occupation of infertile group | Private employee (Ref) |  |  |  |  |  |
|  | Daily laborer | -7.607 (-10.297,-4.917) | -0.256 | -5.554 | 1.370 | 0.0001 |
|  | Government employee | -0.705 (-1.119,2.530) | 0.035 | 0.759 | 0.929 | 0.448 |
|  | NGO | 7.623 (-1.136,14.109) | 0.094 | 2.308 | 3.303 | 0.021 |
| Types of surgery | Abdominal surgery (Ref) |  |  |  |  |  |
|  | Gynecologic surgery | -12.435(-14.159,-10.711) | -0.506 | -14.163 | 0.878 | <0.0001 |
|  | Other surgery | -1.140 (-5.829,3.547) | -0.017 | -0.471 | 2.387 | 0.633 |
| Types of previous history of gynecologic disease | Uterine disease (Ref) |  |  |  |  |  |
|  | Ovarian disease | -13.080 (-17.374,-8.785) | -0.218 | -5.982 | 2.187 | <0.0001 |
|  | PID | -10.625 (-12.368,-8.883) | -0.437 | -11.976 | 0.887 | <0.0001* |

*B= Unstandardized regression coefficient, β = Standardized regression coefficient, *statistically significant= p≤ 0.05, ref. = Reference category*
